# The chemical defensome of five model teleost fish

**DOI:** 10.1038/s41598-021-89948-0

**Published:** 2021-05-18

**Authors:** Marta Eide, Xiaokang Zhang, Odd André Karlsen, Jared V. Goldstone, John Stegeman, Inge Jonassen, Anders Goksøyr

**Affiliations:** 1 Department of Biological Sciences, University of Bergen, Bergen, Norway; 2 Computational Biology Unit, Department of Informatics, University of Bergen, Bergen, Norway; 3 Department of Molecular Oncology, Institute for Cancer Research, Oslo University Hospital-Radiumhospitalet, Oslo, Norway; 4 Biology Department, Woods Hole Oceanographic Institution, Woods Hole, MA USA

**Keywords:** Cellular signalling networks, Data mining, Gene regulatory networks, Genome informatics, Functional genomics, Genetic variation, Comparative genomics, Genome evolution

## Abstract

How an organism copes with chemicals is largely determined by the genes and proteins that collectively function to defend against, detoxify and eliminate chemical stressors. This integrative network includes receptors and transcription factors, biotransformation enzymes, transporters, antioxidants, and metal- and heat-responsive genes, and is collectively known as the *chemical defensome*. Teleost fish is the largest group of vertebrate species and can provide valuable insights into the evolution and functional diversity of defensome genes. We have previously shown that the xenosensing pregnane x receptor (*pxr*, *nr1i2*) is lost in many teleost species, including Atlantic cod (*Gadus morhua*) and three-spined stickleback (*Gasterosteus aculeatus*), but it is not known if compensatory mechanisms or signaling pathways have evolved in its absence. In this study, we compared the genes comprising the chemical defensome of five fish species that span the teleosteii evolutionary branch often used as model species in toxicological studies and environmental monitoring programs: zebrafish (*Danio rerio*), medaka (*Oryzias latipes*), Atlantic killifish (*Fundulus heteroclitus*), Atlantic cod, and three-spined stickleback. Genome mining revealed evolved differences in the number and composition of defensome genes that can have implication for how these species sense and respond to environmental pollutants, but we did not observe any candidates of compensatory mechanisms or pathways in cod and stickleback in the absence of *pxr*. The results indicate that knowledge regarding the diversity and function of the defensome will be important for toxicological testing and risk assessment studies.

## Introduction

The aquatic environment is a sink for anthropogenic compounds, and aquatic animals are particularly vulnerable to chemical stressors in their natural habitats. Many of these chemicals may profoundly influence organism health, including viability, growth, performance, and reproductive abilities. Aquatic species are also widely used as model organisms to assess responses to environmental pollutants. In the OECD Guidelines for the Testing of Chemicals, 13 tests for toxic properties of chemicals use fish in general, and often specific fish species such as zebrafish, medaka, Atlantic killifish and three-spined stickleback.

The intrinsic defense against toxic chemicals largely depends on a set of genes and proteins collectively known as the *chemical defensome*^1^. The term “chemical defensome” refers to a wide range of transcription factors, enzymes, transporters, and antioxidant enzymes that together function to detoxify and eliminate harmful compounds, including xenobiotic and endobiotic chemicals. Thus, the composition of genes comprising a species’ chemical defensome will affect the species overall responsiveness and sensitivity towards chemicals stressors. While we believe that all major protein families that contribute to these defenses are included, it is possible that some individual genes (proteins) have been missed. Genomic-scale information is readily gathered, but less readily analyzed to a great depth. The recent years of sequencing efforts have produced high quality genome assemblies from a wide range of species, facilitating genome-wide mapping and annotation of genes. The chemical defensome was first described in the invertebrates sea urchin and sea anemone^1,2^, and was later mapped in zebrafish (*Danio rerio*), coral, arthropods, and partly in tunicates^3–6^. Although these reports show that the overall metabolic pathways involved in the chemical defensome are largely evolutionarily conserved, the detailed comparison of defensome gene composition in different teleost fish species is not studied.

The diversity in both presence and number of gene homologs can vary substantially between fish species due to the two whole genome duplication (WGD) events in early vertebrate evolution^7^ and a third fish-specific WGD event^8^, in addition to other evolutionary mechanisms such as gene loss, inversions and neo- and subfunctionalizations. For example, we have previously shown that several losses of the pregnane x receptor (*pxr*, or *nr1i2*) have occurred independently across teleost evolution^9^. PXR is an important xenosensor and as a ligand-activated transcription factor one of the key regulators of the chemical defensome^10,11^. The importance of PXR in response to chemical stressors in vertebrates, raises questions of how some fish species cope without this gene.

Thus, the objective of this study was to compare the chemical defensome of zebrafish (*Danio rerio*), medaka (*Oryzias latipes*), and Atlantic killifish (*Fundulus heteroclitus*), which are species that have retained a *pxr* gene*,* to Atlantic cod (*Gadus morhua*) and three-spined stickleback (*Gasterosteus aculeatus*), that have lost this gene by independent mechanisms. Genomes of zebrafish, medaka, killifish, and stickleback were chosen based on their widespread use as model species in both developmental and toxicological studies^12–15^. Atlantic cod is an ecologically and economically important species in the North Atlantic Ocean, and has commonly been used as a bioindicator species in environmental monitoring programs^16–18^. The genome of Atlantic cod was first published in 2011^19^, which has facilitated its increased use as model in toxicological studies^20–24^. Although these fish are all benthopelagic species, their natural habitats range from freshwater to marine environments, from tropical to temperate conditions, and reach sizes ranging from less than five cm (zebrafish and medaka) and 15 cm (killifish and stickleback) to 200 cm (Atlantic cod).

Our results include an overview of the full complement of putative chemical defensome genes in these five fish species. Furthermore, by using previously published transcriptomics data, we investigated the transcriptional expression of defensome genes in early development of zebrafish and stickleback, and their responses to the model polycyclic aromatic hydrocarbon contaminant, benzo(a)pyrene.

In conclusion, our study represents the first interspecies comparison of the full complement of chemical defensome genes in teleost model species. We found that although most defensome genes have been retained in the teleost genomes over millions of years, there are distinct differences between the species. Based on our results, we suggest a holistic approach to analyze omics datasets from toxicogenomic studies, where differences in the chemical defensome gene complement are taken into consideration.

## Material and methods

### Sequence resources

The mapping of chemical defensome genes were performed in the most recently published fish genomes available in public databases (Supplementary Table 1, available at FAIRDOMHub: 10.15490/fairdomhub.1.document.925.3). For zebrafish (*Danio rerio,* GRCz11), three-spined stickleback (*Gasterosteus aculatus,* BROAD S1), Atlantic killifish (*Fundulus heteroclitus,* Fundulus_heteroclitus-3.0.2), and Japanese medaka HdrR (*Oryzias latipes,* ASM223467v1), we used the genome assemblies and annotations available in ENSEMBL. For Atlantic cod (*Gadus morhua*), we used the recent gadMor3 genome assembly available in NCBI (GCA_902167405). For all five species, we focused on the protein coding genes and transcripts.

### Identification of chemical defensome genes

The list of genes included in the chemical defensome (available at FAIRDOMHub: https://doi.org/10.15490/fairdomhub.1.datafile.3957.1), were based on the previous publications defining the chemical defensome^1–3^.

Two main approaches were used to identify the genes related to the chemical defensome of the five fish species. First, we searched the current annotations in NCBI for Atlantic cod or ENSEMBL for zebrafish, stickleback, killifish, and medaka using gene names. For the well-annotated zebrafish genome, this approach successfully identified the genes that are part of the chemical defensome, as previously mapped by Stegeman, et al.^3^. Alternative gene sequences, e.g. splice variants, were removed when identifying the number of genes within the different gene families, but included in the RNA-Seq analyses.

However, only relying on annotations will not identify all defensome genes in the other less characterized fish genomes. Thus, secondly, we also performed hidden Markov model (HMM) searches using HMMER and Pfam profiles representing protein families that are part of the chemical defensome (available at FAIRDOMHub: https://doi.org/10.15490/fairdomhub.1.datafile.3956.1) in the genomes of the remaining four fish species. Putative orthologs of the retrieved protein sequences were identified using reciprocal best hit BLAST searches against the well-annotated zebrafish proteome. To capture any species-specific duplications in the fish genomes compared to the zebrafish reference genome, hits from one-way BLAST hits were also included. The identified peptide sequence IDs were subsequently converted to their related gene IDs using the BioMart tool on ENSEMBL (https://m.ensembl.org/biomart/martview) and R package “mygene” (https://doi.org/doi:10.18129/B9.bioc.mygene). Finally, the resulting gene lists were refined to contain only members of gene families and subfamilies related to the chemical defensome, using the same defensome gene lists as in the first approach (https://doi.org/10.15490/fairdomhub.1.datafile.3957.1).

### Transcription of chemical defensome genes in early development

No embryos were directly used in this study. RNA-Seq datasets of early developmental stages of zebrafish (expression values in Transcripts Per Million from ArrayExpress: E-ERAD-475) and stickleback (sequencing reads from NCBI BioProject: PRJNA395155) were previously published by White, et al.^25^ and Kaitetzidou, et al.^26^, respectively.

For stickleback, embryos were sampled at early morula, late morula, mid-gastrula, early organogenesis, and 24 h post hatching (hph). The sequencing data was processed and analyzed following the automatic pipeline RASflow^27^. Briefly, the reads were mapped to the stickleback genome downloaded from ENSEMBL of version release-100. HISAT2^28^ was used as aligner and featureCounts^29^ was used to count the reads. The library sizes were normalized using Trimmed Mean of M values (TMM)^30^ and the Counts Per Million (CPM) were calculated using R package edgeR^31^. The source code and relevant files can be found on GitHub: https://github.com/zhxiaokang/fishDefensome/tree/main/developmentalStages/stickleback/RASflow.

The zebrafish dataset included 18 time points from one cell to five days post fertilization (dpf). In order to best compare to the available stickleback developmental data, we chose to include the following time points in this study: Cleavage_2 cell (early morula), blastula_1k cell (late morula), mid-gastrula, segmentation_1-4-somites (early organogenesis), and larval_protruding_mouth (24 hph).

The temporal clustering was performed on both species. The R package “tscR”^32^, which clusters time series data using slope distance was applied here, to account for the gene expression variation pattern over time, instead of the absolute gene expression values.

### Exposure response of defensome genes in zebrafish early development

RNA-Seq datasets of zebrafish exposed to benzo(a)pyrene (B(a)P) (gene counts from NCBI GEO: GSE64198) were previously published by Fang, et al.^33^. Briefly, the datasets are results from the following in vivo experiments: Adult parental zebrafish were waterborne-exposed to 50 μg/L B(a)P or 0.1 mL/L ethanol vehicle control, and their eggs collected from days 7 to 11. The eggs were further raised in control conditions or continuously exposed to 42.0 ± 1.9 μg/L B(a)P until 3.3 and 96 (hours post fertilization, hpf), where mid-blastula state and complete organogenesis is reached, respectively. At 3.3 or 96 hpf, embryos (50/pool) or larvae (15/pool) were pooled for RNA isolation, giving three replicate groups of control and exposed at 3.3 hpf and two replicate groups of control and exposed at 96 hpf.

The RNA-Seq reads were mapped to the zebrafish genome and the genes were quantified in the original publication by Fang, et al.^33^. In our study, the gene counts were then normalized followed by differential expression analysis using edgeR^31^ and the chemical defensome genes were then identified based on our defensome gene list of zebrafish.

## Results and discussion

### Chemical defensome genes present in model fish genomes.

**Figure 1:**
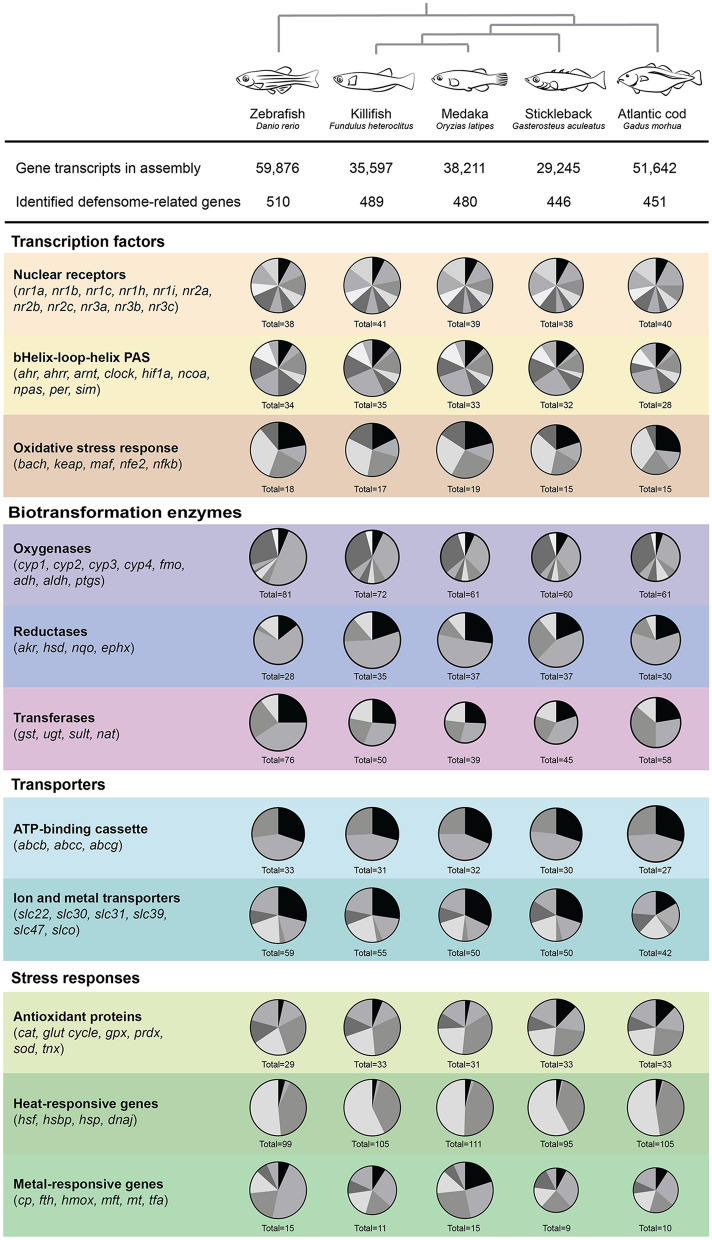
Chemical defensome genes in five model fish species. The genes were identified by searching gene names and using HMMER searches with Pfam profiles, followed by reciprocal or best-hit blast searches towards the zebrafish proteome. The gene families are organized in categories following Gene Ontology annotations and grouped by their role in the chemical defensome. The size of the disk represents the relative number of genes in the different fish genomes within each group, with the number of genes in a specific gene family as slices.

The full complement of chemical defensome genes in zebrafish, killifish, medaka, stickleback and Atlantic cod are available at the FAIRDOMHub (https://doi.org/10.15490/fairdomhub.1.datafile.3958.1). In short, genome analyses of the selected fish species show that the number of chemical defensome genes range from 446 in stickleback to 510 in zebrafish (Fig. 1). Although the number of putative homologous genes in each subfamily varies, we found that all gene subfamilies of the chemical defensome is represented in each species, except for the absence of *pxr* in stickleback and Atlantic cod. While we believe that all major protein families that are related to the chemical defensome are included, we cannot preclude that there are individual genes that are not identified due to the genome quality and level of annotation.

#### Soluble receptors and transcription factors

Stress-activated transcription factors serve as important first responders to many chemicals, and in turn regulate the transcription of other parts of the chemical defensome. Nuclear receptors (NRs) are a superfamily of structurally similar, ligand-activated transcription factors, where members of subfamilies NR1A, B, C, H, and I (such as retinoid acid receptors, peroxisomal proliferator-activated receptors, and liver X receptor), NR2A and B (hepatocyte nuclear factors and retinoid x receptors), and NR3 (such as estrogen receptors and androgen receptor) are involved in the chemical defense^34–37^. All NR subfamilies were found in the five fish genomes, except for the *nr1i2* gene (Supplementary Table 2, available at FAIRDOMHub: 10.15490/fairdomhub.1.document.925.3). We did not find an expansion of any of the groups that indicated an evolved compensational receptor in the absence of *pxr*.

NR1I2, or pregnane x receptor (PXR) is considered an important xenosensor responsible for the transcription of many genes involved in the biotransformation of xenobiotics^10,38^. We have previously shown that loss of the *pxr* gene has occurred multiple times in teleost fish evolution^9^, including in Atlantic cod and stickleback. Interestingly, our searches did not reveal a *pxr* gene in the ENSEMBL genome assembly of Japanese medaka HdRr, which is considered the reference medaka strain^39,40^. In contrast, a *pxr* gene is annotated in the ENSEMBL genomes of the closely related medaka strains HSOK and HNI. Our previous study identified a sequence similar to zebrafish Pxr in the MEDAKA1 (ENSEMBL release 93) genome^9^, and a partial coding sequence (cds) of *pxr* is cloned from medaka genome^41^. However, the specific strain of these resources is not disclosed.

To assess the possible absence of *pxr* in the medaka HdRr strain, we also performed synteny analysis. In vertebrate species, including fish, *pxr* is flanked by the genes *maats1* and *gsk3b*. These genes are also annotated in medaka HdRr, but the specific gene region has a very low %GC and low sequence quality. Thus, we suspect that the absence of *pxr* in the Japanese medaka HdRr genome is due to a sequencing or assembly error. However, until more evidence of its absence can be presented, we chose to include the medaka *pxr* gene (UniProt ID A8DD90_ORYLA) in our resulting list of medaka chemical defensome genes.

Other important transcription factors are the basic helix-loop-helix Per-Arnt-Sim (bHLH/PAS) proteins and the oxidative stress-activated transcription factors. bHLH/PAS proteins are involved in circadian rhythms (such as *clock* and *arntl*), hypoxia response (such as *hif1a* and *ncoa*), development (such as *sim*), and the aryl hydrocarbon receptor pathway (*ahr* and *arnt*) (as reviewed by Gu, et al.^42^ and Kewley, et al.^43^), while oxidative stress-activated transcription factors respond to changes in the cellular redox status and promote transcription of antioxidant enzymes^44,45^. The latter protein family includes nuclear factor erythroid-derived 2 (NFE2), NFE2-like (NFE2L, also known as NRFs) 1, 2, and 3, BACH, the dimerization partners small-Mafs (MafF, MafG and MafK), and the inhibitor Kelch-like-ECH-associated protein 1 (KEAP1)^46^. Putative orthologs for all these subfamilies were identified in all five fish genomes.

#### Biotransformation enzymes

In the first phase of xenobiotic biotransformation, a set of enzymes modifies substrates to more hydrophilic and reactive products. The most important gene family of oxygenases is the cytochrome P450 enzymes (CYPs, EC 1.14.-.-), a large superfamily of heme-proteins that initiate the biotransformation of numerous xenobiotic compounds through their monooxygenase activity^47^. The subfamilies considered to be involved in xenobiotic transformation is Cyp1, Cyp2, Cyp3, and Cyp4, and genes of these families were found in all fish species. Interestingly, our results show that the zebrafish genome holds almost twice as many genes belonging to the *cyp2* subfamily compared to the other four fish species. This is in line with previous findings comparing the CYPome in zebrafish to that in human and cod^48,49^, but the reason for the *cyp2* gene bloom in zebrafish remains unknown. The number of genes in each subfamily differs slightly from previous mappings of the CYPome of zebrafish and cod^48,49^ (Supplementary Table 3, available at FAIRDOMHub: 10.15490/fairdomhub.1.document.925.3). This is likely explained by the sequence and annotation improvement in latest genome assemblies.

Other oxygenases include flavin-dependent monooxygenases (FMOs, EC 1.14.13.8), aldehyde dehydrogenases (ALDH, EC 1.2.1.3), alcohol dehydrogenases (ADHs, EC 1.1.1.1), and prostaglandin-endoperoxide synthases (PTGS, also known as cyclooxygenases, EC 1.14.99.1). Of these, *aldh* represented the largest family in our study, with number of putative gene orthologs ranging from 19 to 22 genes. In comparison, *fmo* had only one gene in zebrafish and four in killifish and cod.

**Figure 2:**
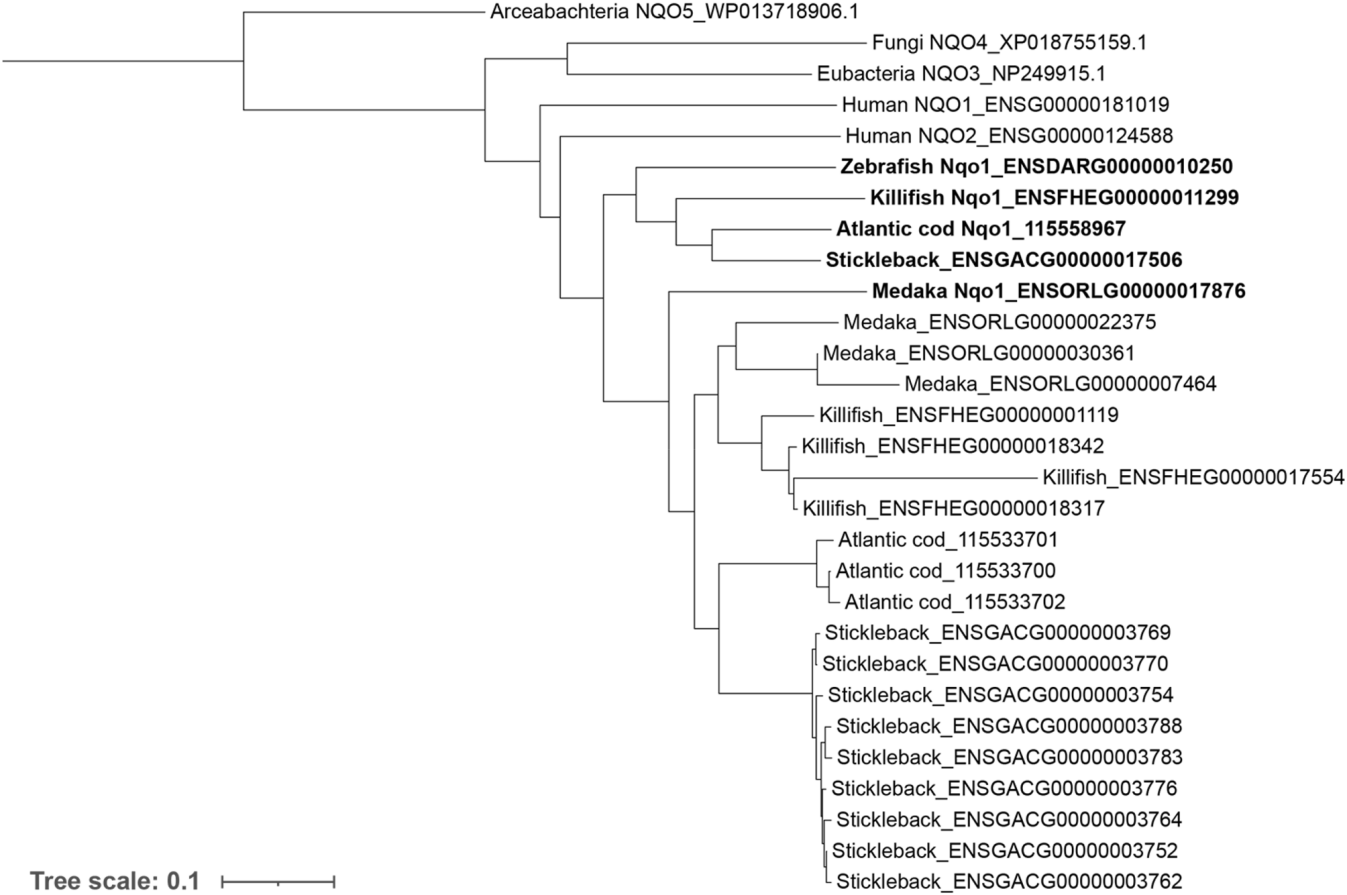
Phylogenetic tree of NAD(P)H:quinone oxidoreductases (NQO), also known as DT-diaphorase (DTD). Multiple sequence alignment and phylogenetic tree was built using Clustal Omega^50^ with standard settings. The tree was drawn using iTol^51^, and rooted with the archaebacterial NQO5.

Furthermore, reductases modify chemicals by reducing the number of electrons. Reductases include aldo–keto reductases (AKRs, EC 1.1.1), hydroxysteroid dehydrogenases (HSDs, EC 1.1.1), epoxide hydrolases (EPHXs, EC 3.3.2.9 and EC 3.3.2.10), and the NAD(P)H:quinone oxidoreductases (NQOs, EC 1.6.5.2). Interestingly, the number of putative orthologous genes in the *nqo* reductase families varied greatly between the fish species, ranging from one in zebrafish to ten in stickleback. A phylogenetic analysis of the evolutionary relationship of the sequences (Fig. 2), shows that all fish species have a Nqo1 annotated gene. In addition, medaka, killifish, stickleback and cod have three to nine other closely related genes. The endogenous functions of the different *nqo* genes found in fish, and thus the consequences of their putative evolutionary gain in teleost fish, remains unknown and should be studied further.

In the second phase of biotransformation, endogenous polar molecules are covalently attached to xenobiotic compounds by transferases, thus facilitating the generation of more water-soluble products that can be excreted from the cells and the organism. An important class of such conjugating enzymes are glutathione S-transferases (GSTs, EC 2.5.1.18), which are divided into three superfamilies: the cytosolic GSTs (divided in six subfamilies designated alpha through zeta), the mitochondrial GST (GST kappa) and the membrane-associated GST (designated MAPEG)^52,53^. Other classes of conjugating enzymes in vertebrates include cytosolic sulfotransferases (SULTs, EC 2.8.2), UDP-glucuronosyl transferases (UGTs, EC 2.4.1.17), N-acetyltransferases (NATs, EC 2.3.1.5), and arylamine NATs (aa-NAT).

Our searches identified a *gstp* gene in zebrafish and cod genomes, but not in medaka, killifish and stickleback. Gstp is previously identified as the major Gst isoenzyme in livers of marine salmonid species^54^. Although the specificity of GSTP is not fully understood, its activity seems related to oxidative stress^55^. Furthermore, the total number of *gst* encoding genes was substantially higher in zebrafish (19 genes) compared to the other fish species (9 in stickleback, 10 in medaka, and 13 in killifish and in cod).

Similarly, the number of *ugt* encoding genes is considerably higher in zebrafish compared to the other fish genomes. Whereas 31 *ugt* genes were found annotated in zebrafish, we only identified 15 in killifish, 11 genes in medaka, 17 in stickleback, and 16 in cod. All Ugt subfamilies (ugt1, -2, -5, and -8) are represented in the different fish genomes but include a varying number of homologs. Previous publications indicate that the number of zebrafish *ugt* encoding genes are as high as 45, with the ugt5 subfamily only existing in teleost and amphibian species^56^. One study has found cooperation of NQO1 and UGT in detoxification of vitamin K3 in HEK293 cell line^57^. However, it is not known if there is any correlation between the high number of *ugt* genes and low number of *nqo* genes in zebrafish, relative to the other fish species.

#### Transporter proteins

Energy-dependent efflux transport of compounds across both extra- and intracellular membranes is facilitated by ATP-binding cassette (ABC) transporters. In humans, the ABCs are organized into seven subfamilies, named ABC A through G, where proteins of B (also known as MDR1), C and G are known to be involved in multidrug resistance (MDR)^58^. Our results showed that the number of *abc* genes in each subfamily were quite similar between the fish, except for Atlantic cod that showed a lower number of *abcb* and *abcc* compared to the other species (Supplementary Table 4, available at FAIRDOMHub: 10.15490/fairdomhub.1.document.925.3). *Abcc1*, also known as multidrug resistance-associated protein (*mrp1*) were identified in all species, whereas *abcb1*, also known as multidrug resistance protein (*mdr1*), were only observed in killifish. Neither was *abcb5*, which is suggested as a close ortholog to *abcb1* in zebrafish, found in medaka, stickleback and cod, which is in line with previous results^59^. A separate group of proteins is called the Solute Carrier (SLC) ‘superfamily,’ which consists of diverse non-homologous groups of ion and metal transporting membrane proteins that facilitate passive transport^60^. Relevant solute carrier proteins include the drug transporting SLC22 and SLC47, the zinc transporting SLC30 and SLC39, the copper transporting SLC31, and the organic anion transporting SLCO^61^.

We found that the number of *abcb*, *abcc*, *abcg*, *slc*, and *slco* genes was similar between the fish species, with zebrafish holding a slightly higher number of homologs. MDR and P-glycoproteins have been relatively understudied in fish^62–64^. A clade of *abch* transporters related to *abcg* is found in some fish species, including zebrafish, but not in Japanese medaka, stickleback and cod^65^. However, as the endogenous function of these genes are not determined, we have not included them specifically into this study.

#### Antioxidant proteins

Antioxidant proteins protect against harmful reactive oxygen species (ROS), such as superoxide anions, hydrogen peroxide and hydroxyl radicals that are formed as by-products in many physiological processes^66,67^. The enzyme superoxide dismutase (SOD, EC 1.15.1.1) catalyze the conversion of superoxide, one of the most abundant ROS species, to hydrogen peroxide^68^. The further detoxification of hydrogen peroxide can be performed by catalases (CAT, EC 1.11.1.6) and glutathione peroxidases (GPXs, EC 1.11.1.9)^66^. The antioxidants also include the glutathione (GSH) system, where GSH is supplied by reduction of gluthatione disulphide by glutathione reductase (GSR, EC 1.8.1.7), or by *de novo* synthesis via glutamate cysteine ligase (made up by the subunits GCLC and GCLM, EC 6.3.2.2), and glutathione synthase (GSS, EC 6.3.2.3).

Together with xenobiotic metabolizing enzymes, induction of genes and enzymatic activity involved in antioxidant defense has long been recognized as a gold standard in the biomarker approach to environmental studies^69^. Putative orthologs for all antioxidant genes were clearly identified in the five fish genomes examined.

#### Heat-responsive genes

Heat-responsive genes represent the largest functional group of genes in the chemical defensome and act in response to a wide range of endogenous and exogenous stressors, such as temperature-shock and heavy metal exposure^70^. In response to stressors, heat shock factors (HSFs) regulate transcription of heat shock proteins (HSPs)^71^. HSPs are divided into families based on their molecular size, and each subfamily has various cellular tasks, including cytoskeleton modulation, protein folding, and chaperone functioning^72,73^.

Not much is known about the heat shock protein expression in fish^70^. We found that all fish genomes hold putative orthologs of *hsf*, heat shock binding proteins (*hsbp*) and *hsp*. The number of putative orthologs of *hsp* and *dnaj* (formerly known as *hsp40*) is high in all fish genomes, with the highest number in killifish with 40 and 60 genes, respectively.

#### Metal-responsive genes

In response to heavy metals such as zinc, cadmium, and copper, metal-responsive transcription factors (MTFs) induce expression of metal-binding proteins, such as metallothioneins (MT), ferritin (ferritin heavy subunit *fth*/*fthl*); heme oxygenases (*hmox,* EC 1.14.99.3,*),* transferrins (*tfa*), and ferroxidase (also known as ceruloplasmin, *cp*; EC 1.16.3.1)^74^.

In our study, we found putative orthologs for all gene families, except a *mt* encoding gene in stickleback or cod genome assemblies. Metallothioneins are cysteine-rich, low molecular weight proteins, and can thus be lost due to low-quality sequence and subsequent assembly. In discrepancy with the genome data, Mts are previously described in both Atlantic cod (Hylland et al. 1994) and stickleback (Uren Webster 2017), and these protein IDs were included into our overview. Similarly, only one or two *mt* genes were found in zebrafish, killifish, and medaka, and this low number is in line with previous findings on metallothioneins in fish^75^.

### Expression of defensome genes during early development of fish

**Figure 3:**
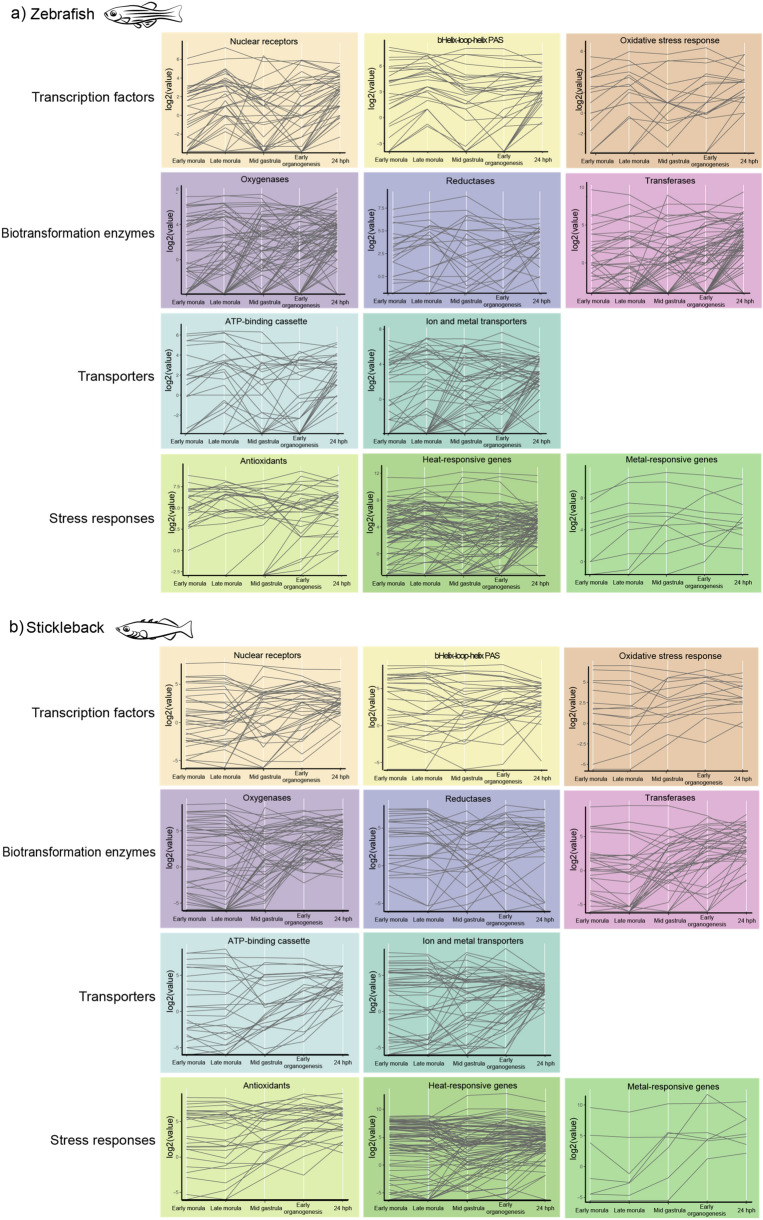
Transcription of chemical defensome genes in early development of (**a**) zebrafish (*Danio rerio*) and (**b**) stickleback (*Gasterasteus aculeatus*). Absolute transcription values (log2 scale) of defensome genes, grouped into their functional category, are shown at early morula, late morula, mid gastrula, early organogenesis, and 24 h post hatching (hph).

The developmental stage at which a chemical exposure event occurs greatly impacts the effect on fish. In general, chemical exposures during early developmental stages of fish cause the most adverse and detrimental effects. Based on data from the ECETOC Aquatic Toxicity database, fish larvae are more sensitive to substances than embryos and juveniles^76^. However, it is not known how the sensitivity is correlated to the expression of the chemical defensome. As examples in this study, we mapped the expression of the full complement of chemical defensome genes during early development using previously published transcriptomics data from zebrafish^25^ and stickleback^26^ (Fig. 3, relevant data available on FAIRDOMHub: https://doi.org/10.15490/fairdomhub.1.assay.1379.1).

Our results showed that many defensome genes are not expressed until after hatching in both species. The delayed genes belonged to all functional categories but were especially prominent where there are several paralogs within the same gene subfamily, for example the transporters and the transferases (Fig. 3). In contrast, the heat shock proteins *hspa8* and *hsp90ab1* were highly transcribed at all stages in both zebrafish and stickleback (Fig. 3). *Hspa8* is a constitutively expressed member of the Hsp70 subfamily, which is previously known as important in rodent embryogenesis^77^. The role of *Hsp90ab1* in development is less known^78^.

Using temporal clustering analysis (relevant data available on FAIRDOMHub: https://doi.org/10.15490/fairdomhub.1.assay.1379.2), we further investigated whether the absence of *pxr* in stickleback lead to a shift in target gene expression during development. Our results did not find clustering patterns of genes related to *pxr,* or any other transcription factor, in neither fish. *Cyp3a65*, a well known zebrafish Pxr target gene^79^, were transcribed at the late morula stage in stickleback and at the mid gastrula stage in zebrafish. This difference could possibly be explained by the difference in synteny of *cyp3a65* in zebrafish and *cyp3a65* in a number of fish species^49^. In a previous study, zebrafish *cyp3a65* appear to have bimodal expression^49^, but this was not observed in the set of developmental stages included in our study. For the glutathione transferases, we found substantial variations in expression between different genes, which is also previously shown at the protein expression and enzyme activity level^80,81^. Thus, although it has been demonstrated that genes regulated by common transcription factors tend to be located spatially close in the genome sequence and thus facilitate a concerted gene expression^82^, we did not observe such patterns in our datasets.

### Exposure response of the defensome genes

**Figure 4:**
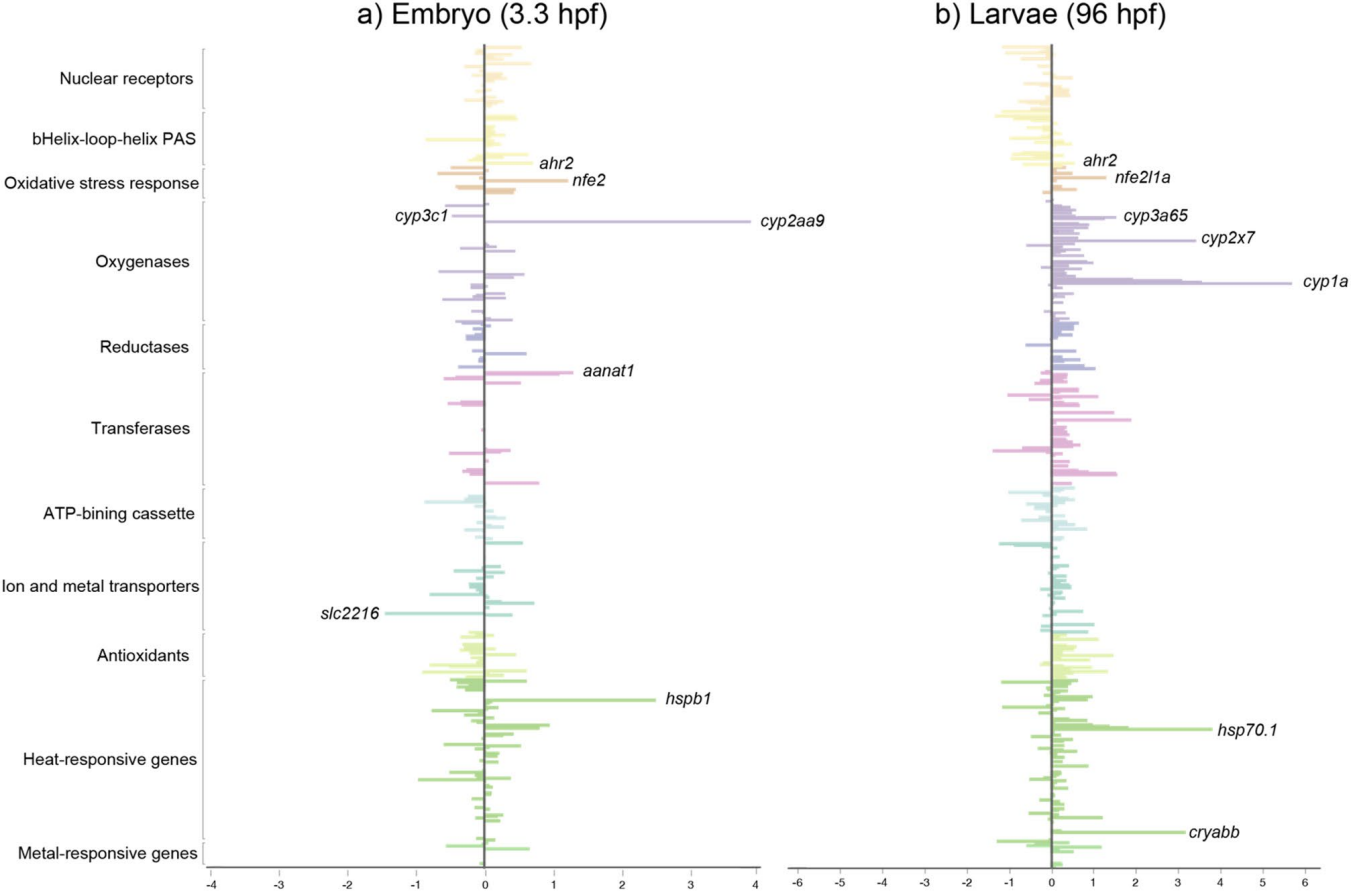
Transcriptional responses on chemical defensome genes in (**a**) zebrafish embryo (3.3 h post fertilization (hpf)) and (**b**) zebrafish larvae (96 hpf) following exposure to benzo(a)pyrene. The transcription is shown as log2 fold change between exposed and control group at each timepoint. The genes are grouped into their functional categories in the chemical defensome and the name of some genes are indicated for clarity.

Next, we studied the transcriptional effect of waterborne exposure to 42.0 ± 1.9 μg/L benzo(a)pyrene (BaP), a well-known Ahr agonist, on the chemical defensome genes at embryonic and larval stages of zebrafish (relevant data available on FAIRDOMHub: https://doi.org/10.15490/fairdomhub.1.datafile.3961.1). In the original publication of the RNA-Seq data, Fang, et al.^33^ showed that the number of differentially expressed genes increased from 8 at the embryonic stage (3.3. hpf) to 1153 at the larvae stage (96 hpf), where the affected pathways included the Ahr detoxification pathway. Focusing on the chemical defensome genes, we show that following exposure at the larval stage, but not embryonic stage, we found a trend of clustered regulation of functionally grouped genes (Fig. 4). In general, transcription factors were downregulated, whereas biotransformation enzymes were upregulated. However, the BaP xenosensor, *ahr2*, and the oxidative stress-responsive transcription factor *nfe2l1a*, were both upregulated (1.4- and 2.4-fold, respectively). The crosstalk between these transcription factors following exposure to chemical stressors is previously studied in zebrafish^83,84^. Furthermore, we showed that at the embryonic stage the exposure led to a strong upregulation of *cyp2aa9* (14-fold) and an increased transcription of single genes such as *ahr2*, *nfe2*, *aanat1*, and *hspb1* (Fig. 4a). These effects were not identified using the differential expression analysis in the original study but can indicate low level changes in the chemical defensome signaling network. *Cyp1a*, which is an established biomarker of exposure to BaP and other polycyclic aromatic hydrocarbons (PAH) in fish^85–87^, was not induced at this stage. However, as described in the original study^33^, we found a strong induction of *cyp1a* (50-fold) at the larvae developmental stage (Fig. 4b). Induction of zebrafish *cyp1a* is previously shown from 24 hpf following exposure to the Ahr model-agonist TCDD^88^.

## Summary and perspectives

The chemical defensome is essential for detoxification and subsequent clearance of xenobiotic compounds, and the composition of the defensome can determine the toxicological responses to many chemicals. Based on the previous annotations of the chemical defensome in other species, our results showed that the number of chemical defensome genes ranged from 446 in three-spined stickleback to 510 in zebrafish due to a varying number of gene homologs in the evolutionarily conserved modules. Of the five fish included in this study, zebrafish has the highest number of gene homologs in most gene families, with the interesting exception of the *nqo* reductases where medaka, killifish, cod, and especially stickleback, had retained a higher number of homologs compared to only one in zebrafish.

We have previously shown that the stress-activated receptor *pxr* gene has been lost in stickleback and cod, but is retained in zebrafish, Atlantic killifish and medaka^9^. The main objective of this study was to explore whether other compensatory genes or signaling pathways had evolved in its absence. However, based on our results, we did not observe such compensatory mechanisms. Still, there could be other variables linked to the absence of *pxr* that fell outside the scope of this study, such as sequence divergences, regulatory elements and expression variation.

Furthermore, we analyzed the transcriptional levels of the defensome genes in early development of zebrafish and stickleback. Importantly, the full complement of defensome genes was not transcribed until after hatching. Comparing the transcriptional effects of waterborne exposure to BaP, a well-known Ahr agonist, on chemical defensome genes at two developmental stages of zebrafish, we found a trend for clustering of gene functional groups at the larvae, but not embryonic, stage.

This study presents characterization of the chemical defensome in five different fish species and at different developmental stages as a way of illustrating and understanding genome-based interspecies and stage-dependent differences in sensitivity and response to chemical stressors. As this study has focused on the presence or absence of chemical defensome genes, and the variation across model teleosts, the role of intraspecies, strain-dependent variants in defensome genes were not included. Several studies have identified defensome gene variants linked to pollution tolerance in fish populations, e.g. in the Ahr pathway^89–92^. In the study by Lille-Langøy, et al.^93^, we previously showed that single-nucleotide polymorphisms (SNPs) in the zebrafish *pxr* gene affect ligand activation patterns. Thus, sequence variation and variation in expression and inducibility also play important roles in chemical sensitivity differences between species, and SNP and copy number variation contributes to sensitivity differences within species.

Traditionally, studying single molecular biomarkers of exposure has proven very useful in toxicological studies^69,94^. Now, the recent advances in omics technologies enable a more holistic view of toxicological responses, including gene set enrichment analysis and pathway analysis approaches^95–98^. However, these analyses can be challenging when working with less studied and annotated species, such as marine teleosts. As seen from our results, studying the full gene complement of the chemical defense system can identify trends of grouped responses that can provide a better understanding of the overall orchestrated effects to chemical stressors, e.g. applied in genome-scale metabolic reconstructions^99^. Such insights will be highly useful in chemical toxicity testing and environmental risk assessment.

## Supplementary information

Supplementary Information.

## References

[1] Goldstone JV (2006). The chemical defensome: Environmental sensing and response genes in the Strongylocentrotus purpuratus genome. Dev. Biol..

[2] Goldstone JV (2008). Environmental sensing and response genes in cnidaria: the chemical defensome in the sea anemone Nematostella vectensis. Cell Biol. Toxicol..

[3] Stegeman, J. J., Goldstone, J. V. & Hahn, M. E. in *Fish physiology: Zebrafish* Ch. 10, (Elsevier, 2010).

[4] Shinzato C, Hamada M, Shoguchi E, Kawashima T, Satoh N (2012). The repertoire of chemical defense genes in the coral Acropora digitifera genome. Zool. Sci..

[5] Yadetie F (2012). Conservation and divergence of chemical defense system in the tunicate Oikopleura dioica revealed by genome wide response to two xenobiotics. BMC Genomics.

[6] De Marco L (2017). The choreography of the chemical defensome response to insecticide stress: insights into the Anopheles stephensi transcriptome using RNA-Seq. Sci. Rep..

[7] Dehal P, Boore JL (2005). Two rounds of whole genome duplication in the ancestral vertebrate. PLoS Biol..

[8] Meyer A, Van de Peer Y (2005). From 2R to 3R: evidence for a fish-specific genome duplication (FSGD). BioEssays.

[9] Eide M (2018). Independent losses of a xenobiotic receptor across teleost evolution. Sci. Rep..

[10] Blumberg B (1998). SXR, a novel steroid and xenobiotic-sensing nuclear receptor. Genes Dev..

[11] Kliewer SA, Goodwin B, Willson TM (2002). The nuclear pregnane X receptor: a key regulator of xenobiotic metabolism. Endocr. Rev..

[12] Hill AJ, Teraoka H, Heideman W, Peterson RE (2005). Zebrafish as a model vertebrate for investigating chemical toxicity. Toxicol. Sci..

[13] Embry MR (2010). The fish embryo toxicity test as an animal alternative method in hazard and risk assessment and scientific research. Aquat. Toxicol..

[14] Burnett KG (2007). Fundulus as the premier teleost model in environmental biology: Opportunities for new insights using genomics. Comp. Biochem. Phys. D.

[15] Katsiadaki I, Scott AP, Mayer I (2002). The potential of the three-spined stickleback (Gasterosteus aculeatus L.) as a combined biomarker for oestrogens and androgens in European waters. Mar. Environ. Res..

[16] Hylland K (2008). Water column monitoring near oil installations in the North Sea 2001–2004. Mar. Pollut. Bull..

[17] Brooks SJ (2011). Water column monitoring of the biological effects of produced water from the ekofisk offshore oil installation from 2006 to 2009. J. Toxicol. Environ. Heal. A.

[18] Holth TF, Beylich BA, Camus L, Klobucar GI, Hylland K (2011). Repeated sampling of Atlantic cod (Gadus morhua) for monitoring of nondestructive parameters during exposure to a synthetic produced water. J. Toxicol. Environ. Health A.

[19] Star B (2011). The genome sequence of Atlantic cod reveals a unique immune system. Nature.

[20] Yadetie F (2018). RNA-Seq analysis of transcriptome responses in Atlantic cod (Gadus morhua) precision-cut liver slices exposed to benzo[a]pyrene and 17 alpha-ethynylestradiol. Aquat. Toxicol..

[21] Yadetie F, Karlsen OA, Eide M, Hogstrand C, Goksøyr A (2014). Liver transcriptome analysis of Atlantic cod (Gadus morhua) exposed to PCB 153 indicates effects on cell cycle regulation and lipid metabolism. BMC Genomics.

[22] Bizarro C, Eide M, Hitchcock DJ, Goksøyr A, Ortiz-Zarragoitia M (2016). Single and mixture effects of aquatic micropollutants studied in precision-cut liver slices of Atlantic cod (Gadus morhua). Aquat. Toxicol..

[23] Hansen BH (2019). Embryonic exposure to produced water can cause cardiac toxicity and deformations in Atlantic cod (Gadus morhua) and haddock (Melanogrammus aeglefinus) larvae. Mar. Environ. Res..

[24] Eide M, Karlsen OA, Kryvi H, Olsvik PA, Goksøyr A (2014). Precision-cut liver slices of Atlantic cod (Gadus morhua): An in vitro system for studying the effects of environmental contaminants. Aquat. Toxicol..

[25] White RJ (2017). A high-resolution mRNA expression time course of embryonic development in zebrafish. Elife.

[26] Kaitetzidou E, Katsiadaki I, Lagnel J, Antonopoulou E, Sarropoulou E (2019). Unravelling paralogous gene expression dynamics during three-spined stickleback embryogenesis. Sci. Rep..

[27] Zhang XK, Jonassen I (2020). RASflow: an RNA-Seq analysis workflow with Snakemake. BMC Bioinform..

[28] Kim D, Landmead B, Salzberg SL (2015). HISAT: a fast spliced aligner with low memory requirements. Nat Methods.

[29] Liao Y, Smyth GK, Shi W (2014). featureCounts: an efficient general purpose program for assigning sequence reads to genomic features. Bioinformatics.

[30] Robinson MD, Oshlack A (2010). A scaling normalization method for differential expression analysis of RNA-seq data. Genome Biol..

[31] Robinson MD, McCarthy DJ, Smyth GK (2010). edgeR: a Bioconductor package for differential expression analysis of digital gene expression data. Bioinformatics.

[32] tscR: A time series clustering package combining slope and Frechet distances. v. R package version 1.2.0. (Bioconductor version: Release (3.12), 2020).

[33] Fang XF (2015). Transcriptomic Changes in Zebrafish Embryos and Larvae Following Benzo[a]pyrene Exposure. Toxicol. Sci..

[34] Mangelsdorf DJ (1995). The nuclear receptor superfamily - the 2nd decade. Cell.

[35] Jin LH, Li Y (2010). Structural and functional insights into nuclear receptor signaling. Adv. Drug Deliv. Rev..

[36] Aranda A, Pascual A (2001). Nuclear hormone receptors and gene expression. Physiol. Rev..

[37] Bertrand S (2007). Unexpected novel relational links uncovered by extensive developmental profiling of nuclear receptor expression. Plos Genet..

[38] Kretschmer XC, Baldwin WS (2005). CAR and PXR: xenosensors of endocrine disrupters?. Chem. Biol. Interact..

[39] Spivakov M (2014). Genomic and phenotypic characterization of a wild medaka population: towards the establishment of an isogenic population genetic resource in fish. G3.

[40] Kirchmaier S, Naruse K, Wittbrodt J, Loosli F (2015). The genomic and genetic toolbox of the teleost medaka (Oryzias latipes). Genetics.

[41] Milnes MR (2008). Activation of steroid and xenobiotic receptor (SXR, NR1I2) and its orthologs in laboratory, toxicologic, and genome model species. Environ. Health Perspect..

[42] Gu YZ, Hogenesch JB, Bradfield CA (2000). The PAS superfamily: sensors of environmental and developmental signals. Annu. Rev. Pharmacol. Toxicol..

[43] Kewley RJ, Whitelaw ML, Chapman-Smith A (2004). The mammalian basic helix-loop-helix/PAS family of transcriptional regulators. Int. J. Biochem. Cell Biol..

[44] Nguyen T, Sherratt PJ, Pickett CB (2003). Regulatory mechanisms controlling gene expression mediated by the antioxidant response element. Annu. Rev. Pharmacol. Toxicol..

[45] Oyake T (1996). Bach proteins belong to a novel family of BTB-basic leucine zipper transcription factors that interact with MafK and regulate transcription through the NF-E2 site. Mol. Cell. Biol..

[46] Nguyen T, Nioi P, Pickett CB (2009). The Nrf2-antioxidant response element signaling pathway and its activation by oxidative stress. J. Biol. Chem..

[47] Nelson DR (2004). Comparison of cytochrome P450 (CYP) genes from the mouse and human genomes, including nomenclature recommendations for genes, pseudogenes and alternative-splice variants. Pharmacogenetics.

[48] Karlsen OA, Puntervoll P, Goksøyr A (2012). Mass spectrometric analyses of microsomal cytochrome P450 isozymes isolated from beta-naphthoflavone-treated Atlantic cod (Gadus morhua) liver reveal insights into the cod CYPome. Aquat. Toxicol..

[49] Goldstone JV (2010). Identification and developmental expression of the full complement of Cytochrome P450 genes in Zebrafish. BMC Genom..

[50] Madeira F (2019). The EMBL-EBI search and sequence analysis tools APIs in 2019. Nucl. Acids Res..

[51] Letunic I, Bork P (2019). Interactive tree of life (iTOL) v4: recent updates and new developments. Nucl. Acids Res..

[52] Hayes JD, Flanagan JU, Jowsey IR (2005). Glutathione transferases. Annu. Rev. Pharmacol. Toxicol..

[53] Nebert DW, Vasiliou V (2004). Analysis of the glutathione S-transferase (GST) gene family. Hum. Genomics.

[54] Dominey RJ, Nimmo IA, Cronshaw AD, Hayes JD (1991). The major glutathione-S-transferase in salmonid fish livers is homologous to the mammalian Pi-class gst. Comp. Biochem. Phys. B.

[55] Tew KD (2011). The role of glutathione S-transferase P in signaling pathways and S-glutathionylation in cancer. Free Radic. Biol. Med..

[56] Huang HY, Wu Q (2010). Cloning and comparative analyses of the zebrafish ugt repertoire reveal its evolutionary diversity. PLoS ONE.

[57] Nishiyama T (2010). Cooperation of NAD(P)H:quinone oxidoreductase 1 and UDP-glucuronosyltransferases reduces menadione cytotoxicity in HEK293 cells. Biochem. Biophys. Res. Commun..

[58] Dean M, Hamon Y, Chimini G (2001). The human ATP-binding cassette (ABC) transporter superfamily. J. Lipid Res..

[59] Luckenbach T, Fischer S, Sturm A (2014). Current advances on ABC drug transporters in fish. Comp. Biochem. Physiol. C Toxicol. Pharmacol..

[60] Hediger MA (2004). The ABCs of solute carriers: physiological, pathological and therapeutic implications of human membrane transport proteins: introduction. Pflug Arch. Eur. J. Phys..

[61] Roth M, Obaidat A, Hagenbuch B (2012). OATPs, OATs and OCTs: the organic anion and cation transporters of the SLCO and SLC22A gene superfamilies. Br. J. Pharmacol..

[62] Fischer S (2013). Abcb4 acts as multixenobiotic transporter and active barrier against chemical uptake in zebrafish (Danio rerio) embryos. BMC Biol..

[63] Jackson JS, Kennedy CJ (2017). Regulation of hepatic abcb4 and cyp3a65 gene expression and multidrug/multixenobiotic resistance (MDR/MXR) functional activity in the model teleost, Danio rerio (zebrafish). Comp. Biochem. Phys. C.

[64] Ferreira M, Costa J, Reis-Henriques MA (2014). ABC transporters in fish species: a review. Front. Physiol..

[65] Jeong CB (2015). Marine medaka ATP-binding cassette (ABC) superfamily and new insight into teleost Abch nomenclature. Sci. Rep..

[66] Droge W (2002). Free radicals in the physiological control of cell function. Physiol. Rev..

[67] Dupre-Crochet S, Erard M, Nuss O (2013). ROS production in phagocytes: why, when, and where?. J. Leukoc. Biol..

[68] Deby C, Goutier R (1990). New perspectives on the biochemistry of superoxide anion and the efficiency of superoxide dismutases. Biochem. Pharmacol..

[69] van der Oost R, Beyer J, Vermeulen NPE (2003). Fish bioaccumulation and biomarkers in environmental risk assessment: a review. Environ. Toxicol. Pharmacol..

[70] Iwama GK, Thomas PT, Forsyth RHB, Vijayan MM (1998). Heat shock protein expression in fish. Rev. Fish Biol. Fish..

[71] Morimoto RI (1993). Cells in stress: transcriptional activation of heat-shock genes. Science.

[72] Basu N (2002). Heat shock protein genes and their functional significance in fish. Gene.

[73] Kampinga HH (2009). Guidelines for the nomenclature of the human heat shock proteins. Cell Stress Chaperones.

[74] Brugnera E (1994). Cloning, chromosomal mapping and characterization of the human metal-regulatory transcription factor Mtf-1. Nucleic Acids Res..

[75] Smirnov LP, Sukhovskaya IV, Nemova NN (2005). Effects of environmental factors on low-molecular-weight peptides of fishes: a review. Russ. J. Ecol..

[76] Hutchinson TH, Solbe J, Kloepper-Sams PJ (1998). Analysis of the ECETOC aquatic toxicity (EAT) database: III: comparative toxicity of chemical substances to different life stages of aquatic organisms. Chemosphere.

[77] Luft JC, Dix DJ (1999). Hsp70 expression and function during embryogenesis. Cell Stress Chaperones.

[78] Haase M, Fitze G (2016). HSP90AB1: Helping the good and the bad. Gene.

[79] Kubota A (2015). Role of pregnane X receptor and aryl hydrocarbon receptor in transcriptional regulation of pxr, CYP2, and CYP3 genes in developing zebrafish. Toxicol. Sci..

[80] Tierbach A, Groh KJ, Schonenberger R, Schirmer K, Suter MJ (2018). Glutathione S-transferase protein expression in different life stages of zebrafish (Danio rerio). Toxicol. Sci..

[81] Tierbach A, Groh KJ, Schonenberger R, Schirmer K, Suter MJ (2020). Biotransformation Capacity of Zebrafish (Danio rerio) Early Life Stages: functionality of the Mercapturic Acid Pathway. Toxicol. Sci..

[82] Zhang J (2019). Spatial clustering and common regulatory elements correlate with coordinated gene expression. PLoS Comput. Biol..

[83] Hahn ME, Timme-Laragy AR, Karchner SI, Stegeman JJ (2015). Nrf2 and Nrf2-related proteins in development and developmental toxicity: insights from studies in zebrafish (Danio rerio). Free Radic. Biol. Med..

[84] Rousseau ME (2015). Regulation of Ahr signaling by Nrf2 during development: effects of Nrf2a deficiency on PCB126 embryotoxicity in zebrafish (Danio rerio). Aquat. Toxicol..

[85] Goksøyr A (1985). Purification of hepatic microsomal cytochromes P-450 from ß-naphthoflavone-treated Atlantic cod *(Gadus morhua)*, a marine teleost fish. Biochim. Biophys. Acta.

[86] Goksøyr A, Förlin L (1992). The cytochrome P450 system in fish, aquatic toxicology, and environmental monitoring. Aquat. Toxicol..

[87] Stegeman JJ, Lech JJ (1991). Cytochrome-P-450 monooxygenase systems in aquatic species: carcinogen metabolism and biomarkers for carcinogen and pollutant exposure. Environ. Health Perspect..

[88] Andreasen EA (2002). Tissue-specific expression of AHR2, ARNT2, and CYP1A in zebrafish embryos and larvae: Effects of developmental stage and 2,3,7,8-tetrachlorodibenzo-p-dioxin exposure. Toxicol. Sci..

[89] Wirgin I (2011). Mechanistic basis of resistance to PCBs in Atlantic tomcod from the Hudson River. Science.

[90] Oziolor EM, Bigorgne E, Aguilar L, Usenko S, Matson CW (2014). Evolved resistance to PCB- and PAH-induced cardiac teratogenesis, and reduced CYP1A activity in Gulf killifish (Fundulus grandis) populations from the Houston Ship Channel, Texas. Aquat. Toxicol..

[91] Williams LM, Oleksiak MF (2011). Ecologically and evolutionarily important SNPs identified in natural populations. Mol. Biol. Evol..

[92] Reid NM (2016). The genomic landscape of rapid repeated evolutionary adaptation to toxic pollution in wild fish. Science.

[93] Lille-Langøy R (2019). Sequence variations in pxr (nr1i2) from zebrafish (Danio rerio) strains affect nuclear receptor function. Toxicol. Sci..

[94] Peakall DB (1994). The role of biomarkers in environmental assessment 1: introduction. Ecotoxicology.

[95] Subramanian A (2005). Gene set enrichment analysis: a knowledge-based approach for interpreting genome-wide expression profiles. Proc. Natl. Acad. Sci. U. S. A..

[96] Fabregat A (2017). Reactome pathway analysis: a high-performance in-memory approach. BMC Bioinform..

[97] Martins C, Dreij K, Costa PM (2019). The state-of-the art of environmental toxicogenomics: challenges and perspectives of "omics" approaches directed to toxicant mixtures. Int. J. Environ. Res. Public Health.

[98] Brooks BW (2020). Toxicology advances for 21st century chemical pollution. One Earth.

[99] Hanna EM (2020). ReCodLiver0.9: overcoming challenges in genome-scale metabolic reconstruction of a non-model species. Front. Mol. Biosci..

