# Supplementary material for: The chemical defensome of five model teleost fish
Source: Sci Rep. 2021 May 18;11:10546. doi: 10.1038/s41598-021-89948-0 (PMC8131381; doi:10.1038/s41598-021-89948-0)
Supplement: Supplementary file 1 — Supplementary Information. [file 41598_2021_89948_MOESM1_ESM.docx]

The chemical defensome of fish

Marta Eide^1*^, Xiaokang Zhang^2,3*^, Odd André Karlsen^1^, Jared V. Goldstone^4^, John Stegeman^4^, Inge Jonassen^2^, Anders Goksøyr^1§^

1. Department of Biological Sciences, University of Bergen, Norway
2. Computational Biology Unit, Department of Informatics, University of Bergen, Norway
3. Department of Molecular Oncology, Institute for Cancer Research, Oslo University Hospital-*Radiumhospitalet*
4. Biology Department, Woods Hole Oceanographic Institution, Woods Hole, MA, USA

^*^ The authors contributed equally to the study

^§^ Corresponding author: anders.goksoyr@uib.no

**Table 1: Overview of teleost genome assemblies retrieved from ENSEMBL and NCBI**

| **Species** | **Latin name** | **Genome** | **Gene build release** | **Base pairs** | **Gene transcripts** |
| --- | --- | --- | --- | --- | --- |
| Zebrafish | *Danio rerio* | GRCz11 | Mar 2018 | 1,674,207,132 | 59,876 |
| Three-spined stickleback | *Gasterosteus aculatus* | BROAD S1 | Aug 2006 | 446,627,861 | 29,245 |
| Atlantic killifish (mummichog) | *Fundulus heteroclitus* | GCA_000826765.1 | Jan 2015 | 1,021,898,560 | 35,597 |
| Japanese medaka HdrR  (Southern Japan) | *Oryzias latipes* | ASM223467v1 | Jul 2018 | 734,057,086 | 38,211 |
| Atlantic cod | *Gadus morhua* | gadMor3 | Jul 2019 | 669,966,409 | 51,642 |

**Table 2: Overview of identified genes in the nuclear receptor (nr) subfamilies related to the chemical defensome in zebrafish (Danio rerio), Atlantic killifish (Fundulus heteroclitus), medaka (Oryzias latipes), three-spined stickleback (Gasterosteus aculeatus), and Atlantic cod (Gadus morhua).**

| **Subfamily** | **Gene** | **Zebrafish** | **Killifish** | **Medaka** | **Stickleback** | **Atlantic cod** |
| --- | --- | --- | --- | --- | --- | --- |
| *nr1a1* | *thra* | x | x | x | x | x |
|  |  | x | x | x | x | x |
| *nr1a2* | *thrb* | x | x | x | x | x |
| *nr1b1* | *rara* | x | x | x | x | x |
|  |  | x | x | x |  | x |
|  |  |  | x |  |  | x |
| *nr1b2* | *rarb* |  |  | x |  | x |
| *nr1b3* | *rarg* | x | x | x | x | x |
|  |  | x | x | x | x | x |
|  |  |  | x |  | x | x |
|  |  |  |  |  | x |  |
| *nr1c1* | *ppara* | x | x | x | x | x |
|  |  | x | x | x | x | x |
| *nr1c2* | *ppard* | x | x | x | x | x |
|  |  | x |  |  |  |  |
| *nr1c3* | *pparg* | x | x | x | x | x |
| *nr1h3* | *lxr* | x | x | x | x | x |
| *nr1h4* | *fxr* | x | x | x | x | x |
| *nr1h5* | *fxrb* | x | x | x | x | x |
| *nr1i1* | *vdr* | x | x | x | x | x |
|  |  | x | x | x | x | x |
| *nr1i2* | *pxr* | x | x | x |  |  |
| *nr2a1* | *hnf4a* | x | x | x | x | x |
| *nr2a1* | *hnf4a* |  | x |  |  | x |
| *nr2a2* | *hnf4g* | x | x | x | x | x |
| *nr2a3* | *hnf4b* | x |  |  |  |  |
| *nr2b1* | *rxra* | x | x | x | x | x |
|  |  | x |  |  |  |  |
| *nr2b2* | *rxrb* | x | x | x | x | x |
|  |  | x | x | x | x | x |
| *nr2b3* | *rxrg* | x | x | x | x | x |
| *nr3a1* | *esr1* | x | x | x | x | x |
| *nr3a2* | *esr2* | x | x | x | x | x |
|  |  | x | x | x | x | x |
| *nr3b1* | *esrra* | x | x | x | x | x |
| *nr3b2* | *esrrb* | x | x | x | x | x |
|  |  |  | x | x | x | x |
|  |  |  | x |  |  |  |
|  | *esrrd* | x |  | x |  |  |
| *nr3b3* | *esrrg* | x | x | x | x | x |
|  |  | x | x | x | x | x |
|  |  |  |  |  | x |  |
| *nr3c1* | *gr* | x | x | x | x | x |
|  |  |  | x | x | x | x |
| *nr3c2* | *mr* | x | x | x | x | x |
| *nr3c3* | *pgr* | x | x | x | x | x |
| *nr3c4* | *ar* | x | x | x | x | x |
|  |  |  | x | x | x | x |

**Table 3: Number of cytochrome P450 (cyp) genes related to the chemical defensome identified in zebrafish (Danio rerio) and Atlantic cod (Gadus morhua) in previous and current studies.**

| **Cyp subfamily** | **Zebrafish** | | | **Atlantic cod** | | |
| --- | --- | --- | --- | --- | --- | --- |
|  | **Goldstone et al. 2010** | **Current study** | **Karlsen et al. 2012** | | **Current study** |  |
| **Genome** | Zv6 and Zv7 | GRCz11 | GmG100427 and GmE100215 (EST) | | gadMor3 |  |
| **Cyp1** | 5 | 5 | 5 | | 3 |  |
| **Cyp2** | 47 | 40 | 14 | | 18 |  |
| **Cyp3** | 5 | 4 | 5 | | 4 |  |
| **Cyp4** | 5 | 4 | 3 | | 5 |  |

**Table 4: Overview of identified genes in the ATP-binding cassette (abc) subfamilies related to the chemical defensome in zebrafish (Danio rerio), Atlantic killifish (Fundulus heteroclitus), medaka (Oryzias latipes), three-spined stickleback (Gasterosteus aculeatus), and Atlantic cod (Gadus morhua).**

|  | **Zebrafish** | **Killifish** | **Medaka** | **Stickleback** | **Atlantic cod** |
| --- | --- | --- | --- | --- | --- |
| abcb | 10 | 9 | 10 | 9 | 8 |
| abcc | 14 | 14 | 14 | 14 | 12 |
| abcg | 8 | 8 | 8 | 7 | 7 |
